# Supplementary material for: Vitamin D Status and Indices of Mineral Homeostasis in the Population: Differences Between 25-Hydroxyvitamin D and 1,25-Dihydroxyvitamin D
Source: Nutrients. 2019 Aug 1;11(8):1777. doi: 10.3390/nu11081777 (PMC6722609; doi:10.3390/nu11081777)

Supplementary data

Table S1. Assay variability in blind duplicate measurements of serum calcidiol and serum calcitriol by chemiluminescent assay: mean±SD.

	**within-assay**	**between-assay**
**Serum calcidiol measurements**		
N samples	50	40
N samples/assay	≥ 5	5
Serum calcidiol, ng/mL		
duplicate 1	27.8±14.3	29.5±15.5
duplicate 2	28.6±15.4	29.1±15.1
Mean of duplicates, ng/mL	28.2±14.8	29.3±15.2
Difference between mean and duplicate^		
ng/mL	0.97±1.08	1.22±1.16
% of mean	3.70±3.73	4.17±3.01
**Serum calcitriol measurements**		
N samples	45	42
N samples/assay	≥5	≥ 5
Serum calcitriol, pg/mL		
duplicate 1	51.8±28.6	55.2±32.0
duplicate 2	52.0±28.6	56.4±33.0
Mean of duplicates, pg/mL	51.9±28.6	55.8±32.4
Difference between mean and duplicate^		
pg/mL	1.23±1.11	1.83±1.93
% of mean	2.62±2.37	3.50±1.91

^ without positive or negative signs.

Table S2. Difference among three datasets of serum calcidiol: comparison of data of non-recalibrated chemiluminescent assay and of immunoassay to data of the recalibrated chemiluminescent assay by paired t-test, regression analysis, and correlation analysis: mean±SD, regression coefficients with 95% confidence interval (95%CI), and square correlation coefficient (R^2).

**Calcidiol dataset**	mean±SD	regression coefficients [a]		R^2
Recalibrated chemiluminescent assay	21.6±12.3	y-axis intercept (95%CI)	slope (95%CI)	
Non-recalibrated chemiluminescent assay	19.8±9.4*	-4.27 (-4.4/-4.1)	1.30 (1.29/1.31)	0.992
Immunoassay	18.9±9.5*	0.99 (0.07/1.91)	1.09 (1.04/1.13)	0.711

* P<0.001 by paired t-test; [a] dependent variable = data of recalibrated chemiluminescent assay

Table S3. Descriptive statistics: mean±SD or prevalence.

	**women**	**men**	**men and women**
Number of persons	478	501	979
Age, y	60.0±10.0	59.8±9.6	59.9±9.8
BMI, kg/m^2	28.7±5.5	28.6±4.2	28.7±4.9
eGFR, mL/min x 1.73 m^2	81.5±16.3	84.5±15.6	83.0±16.0
Calcidiol, recalibrated chemiluminescent assay, ng/mL	21.0±12.7	22.1±11.9	21.6±12.3
% with mild-to-moderate deficiency (10-19 ng/mL)	33.5%	39.3%	36.5%
% with severe deficiency (<10 ng/mL)	20.5%	13.2%	16.8%
Calcidiol, non-recalibrated chemiluminescent assay, ng/mL	19.4±9.8	20.3±9.1	19.8±9.4
% with mild-to-moderate deficiency (10-19 ng/mL)	38.3%	44.3%	41.4%
% with severe deficiency (<10 ng/mL)	18.6%	10.8%	14.6%
Calcidiol, immunoassay, ng/mL	17.6±9.1	20.2±9.7	18.9±9.5
% with mild-to-moderate deficiency (10-19 ng/mL)	43.1%	42.7%	42.9%
% with severe deficiency (<10 ng/mL)	21.3%	13.2%	17.2%

Calcitriol, chemiluminescent assay, pg/mL<br>% with deficiency (<18 pg/mL)	37.5±10.9<br>2.7%	38.3±11.8<br>3.4%	37.9±11.4<br>3.1%
Serum calcium, mg/100 mL<br>% with hypocalcemia (≤ 8.6 mg/100 mL)	9.28±0.30<br>0.6%	9.35±0.30<br>1.2%	9.32±0.31<br>0.9%
Serum albumin, g/L	43.1±2.5	43.5±2.6	43.3±2.6
Albumin-normalized serum calcium, mg/100 mL	9.07±0.20	9.12±0.19	9.10±0.20
Serum PTH, pg/mL<br>% with high PTH (≥ 66 pg/mL)	25.4±13.7<br>1.9%	25.9±12.1<br>1.4%	25.6±12.9<br>1.6%
Serum phosphorus, mg/100 mL	3.34±0.61	2.95±0.57	3.14±0.62
Urine calcium/creatinine, mg/g	132±79	99±75	115±79
Urine phosphorus/creatinine, mg/g	514±221	438±193	475±211
% reporting vitamin D supplementation	5.0% (24)	0.4% (2)	2.7% (26)

Conversion factors to mmol/L:.4 for serum calcium; 0.323 for serum phosphorus. One person reported post-surgical hypoparathyroidism.

Table S4. Differences in prevalence of calcidiol deficiency among three datasets of serum calcidiol.

	dataset		
Calcidiol deficiency	Recalibrated chemiluminescent assay	Non-recalibrated chemiluminescent assay	Immunoassay
severe, <10 ng/mL	16.8% (164)	14.6% (143)*	17.2% (168)[ns]
mild-to-moderate, 10-19 ng/mL	36.5% (357)	41.4% (405)*	42.9% (420)*

Comparison to to recalibrated chemiluminescent assay by McNemar test: * P <0.001 ; [ns] not significant (P>0.05).

Table S5. Association of calcidiol deficiency with hypocalcemia and high serum PTH using data of non-recalibrated chemiluminescent assay and of immunoassay: prevalence, odds ratio, and ROC_{AUC} with 95% confidence interval (95%CI).

	**Hypocalcemia[a]**			**High serum PTH[b]**		
	prevalence	odds ratio *(95%CI)*	ROC_{AUC} *(95%CI)*	prevalence	odds ratio *(95%CI)*	ROC_{AUC} *(95%CI)*
**Non-recalibrated chemiluminescent assay**						
with severe deficiency* <10 ng/mL n = 143	1.4%	1.21 *(0.23/6.30)*	0.519[ns] *(0.30/0.74)*	6.3%	9.58 *(2.56/35.9)*	0.756** *(0.61/0.90)*
with mild-to-moderate deficiency* 10-19 ng/mL n = 405	0.5%	0.42 *(0.08/2.19)*	0.400[ns] *(0.20/0.60)*	1.0%	1.42 *(0.32/6.40)*	0.544[ns] *(0.33/0.76)*
without deficiency ≥ 20 ng/mL n = 431	1.2%	1 (ref)		0.7%	1 (ref)	
**Immunoassay**						
with severe deficiency* <10 ng/mL n = 168	1.8%	3.54 *(0.59/21.4)*	0.651[ns] *(0.40/0.90)*	4.2%	5.62 *(1.44/22.0)*	0.703* *(0.54/0.87)*
with mild-to-moderate deficiency* 10-19 ng/mL n = 420	1.0%	1.87 *(0.34/10.3)*	0.575[ns] *(0.35/0.80)*	1.4%	1.87 *(0.47/7.55)*	0.575[ns] *(0.39/0.76)*
without deficiency ≥ 20 ng/mL n = 391	0.5%	1 (ref)		0.8%	1 (ref)	

[a] hypocalcemia = serum calcium ≤ 8.6 mg/100 mL; [b] high PTH = serum PTH ≥ 66 pg/mL; Significance of ROC_{AUC}: [ns] not significant, * p = 0.027, ** P=0.002.

Figure S1. – Mean±SD of calcidiol measurements (upper panel) and of calcitriol measurements (lower panel) in three reference serum sets by date of assay over the period of measurements in the study cohort (n measurements per assay ≥ 3).

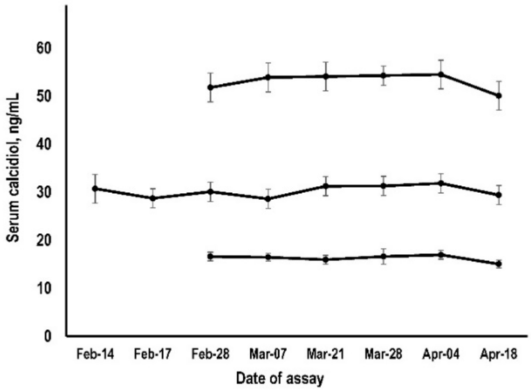

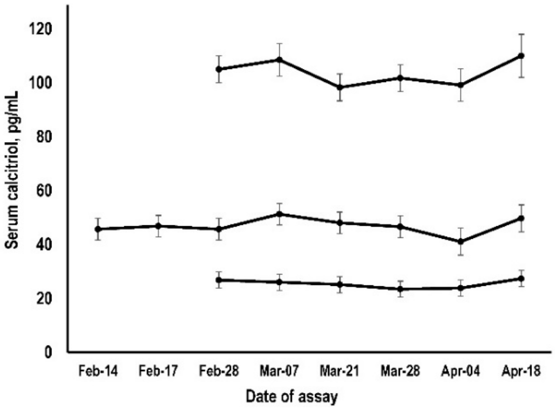

Figure S2. – Certified values of calcidiol concentration in Level 2 and Level 3 of NIST-SRM 972a over measured values of calcidiol using the calibration of the original Diasorin chemiluminescent assay. Mean±95% confidence of the certified value of calcidiol was 18.9±0.4 ng/mL for Level 2 and 33.2±0.5 ng/mL for Level 3. In five different measurements, mean±SE was 18.5±0.5 ng/mL for Level 2 and 29.2±0.5 ng/mL for Level 3 (-2.1% and -12.0% underestimate of the certified values, respectively). The straight line indicates the curve that best fitted the quadratic relation between measured values and certified values ($y = 0.0095x^2 + 0.8563x$; $R^2 = 0.9901$). The dotted line indicates the theoretical identity line.

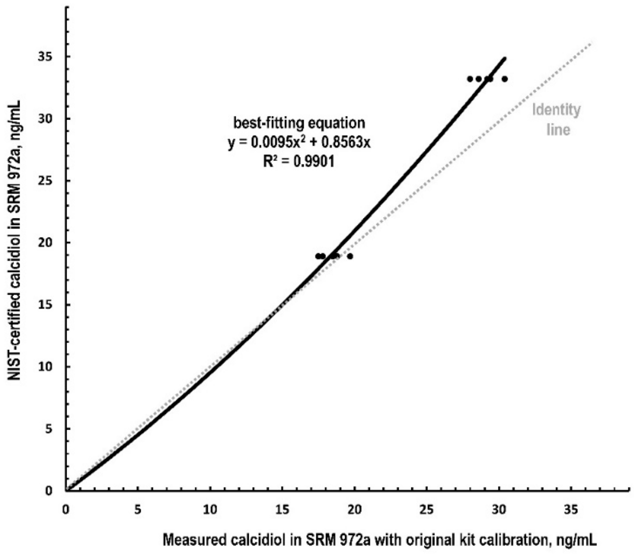

Figure S3. – Frequency distribution and skewness (95% confidence interval, 95%CI) of serum calcidiol measured by non-recalibrated chemiluminescent assay (left panel) and by immunoassay (right panel). The number of persons per stratum of concentration is shown within parentheses on the top of each bar.

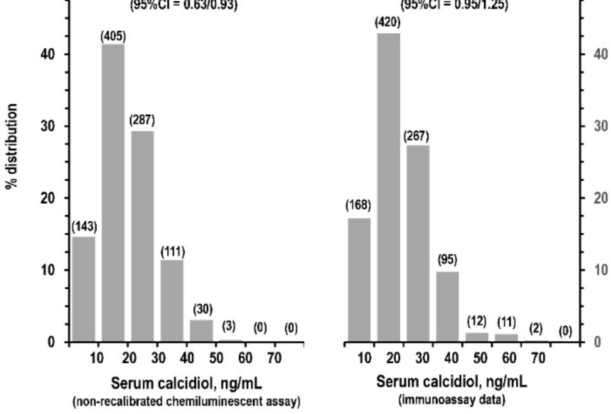

Figure S4. – Mean±SE of albumin-normalized serum calcium[a] by stratum of recalibrated serum calcidiol (left panel) and of serum calcitriol (right panel). Strata were defined as in Figure 1. The stratum with serum calcidiol ≥70 ng/mL and the stratum with serum calcitriol <10 pg/mL were combined with the next stratum to avoid the bias due to low n (n=3). Number of persons per stratum from left to right: for calcidiol = 164, 357, 253, 122, 53, 18, and 12; for calcitriol = 38, 193, 355, 255, 106, 24, and 8. P by non-adjusted ANOVA. P were considered statistically significant when <0.025 to control for double testing (contrast and linear trend). The line shows the linear trend along strata.

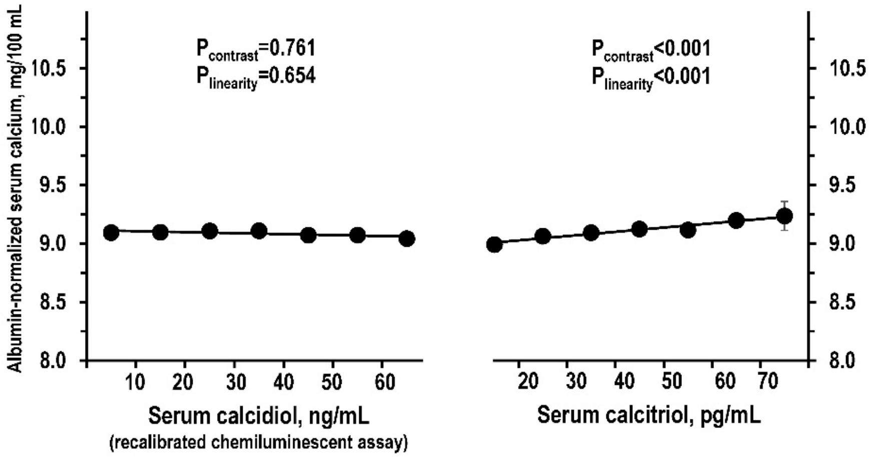

[a] 0.4 mg/100 mL of serum calcium were added or subtracted for every 6 g/L of albumin below or above the reference value of 40 g/L (ref. #9 - Hannan FM, Thakker RV. Investigating hypocalcaemia. *BMJ* 2013; 346: f2213).

Figure S5. Mean±SE of serum calcium (top panels) and of urine calcium/creatinine ratio (lower panels) by stratum of serum calcidiol of non-recalibrated chemiluminescent assay (left panels) and of immunoassay (right panels). Strata were defined as in Figure 1. The stratum with serum calcidiol ≥70 ng/mL and the stratum with serum calcitriol <10 pg/mL were combined with the next stratum to avoid the bias due to low n (n=3). Number of persons per stratum from left to right: for calcidiol = 164, 357, 253, 122, 53, 18, and 12; for calcitriol = 38, 193, 355, 255, 106, 24, and 8. P by non-adjusted ANOVA. P were considered statistically significant when <0.025 to control for double testing (contrast and linear trend). The line shows the linear trend along strata.

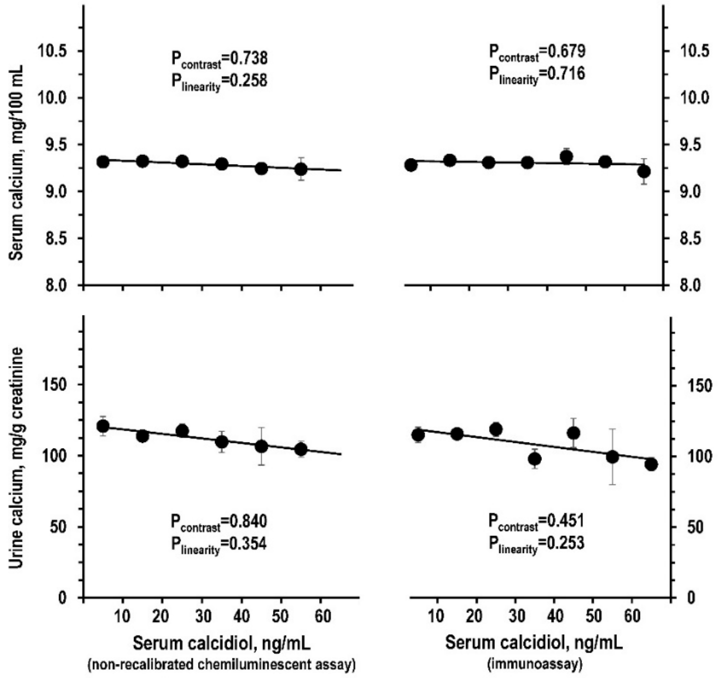

Figure S6. Mean±SE of serum calcium (top panels) and of urine calcium/creatinine ratio (lower panels) by stratum of recalibrated serum calcidiol (left panels) and of serum calcitriol (right panels) with control for other variables (sex, age, BMI, and eGFR). Strata were defined as in Figure 1. The stratum with serum calcidiol ≥70 ng/mL and the stratum with serum calcitriol <10 pg/mL were combined with the next stratum to avoid the bias due to low n (n=3). Number of persons per stratum from left to right: for calcidiol = 164, 357, 253, 122, 53, 18, and 12; for calcitriol = 38, 193, 355, 255, 106, 24, and 8. P by ANOVA. P were considered statistically significant when <0.025 to control for double testing (contrast and linear trend). The line shows the linear trend along strata.

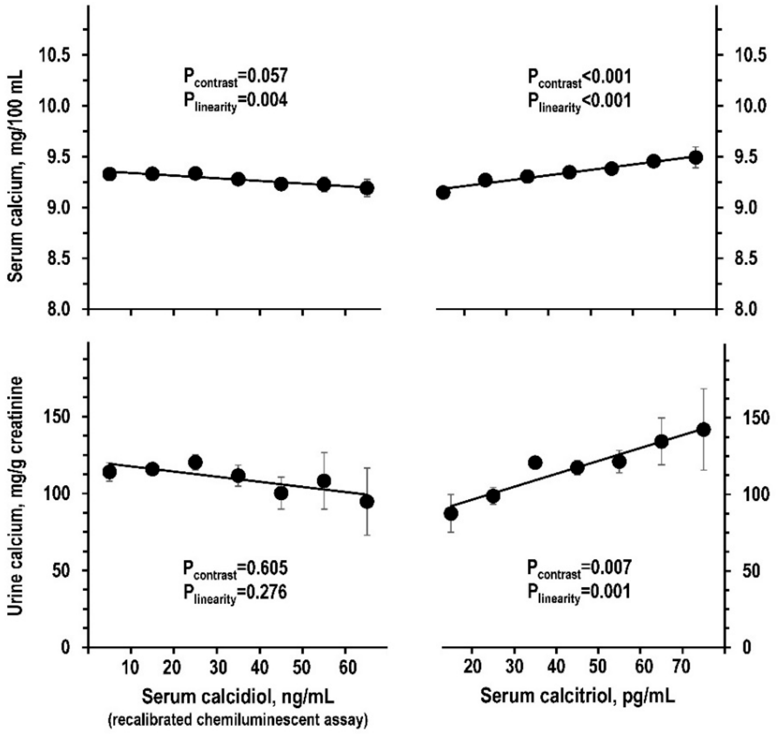

Figure S7. – Mean±SE of serum PTH by stratum of serum calcidiol measured by non-recalibrated chemiluminescent assay (left panel) and by immunoassay (right panel). Strata were defined as in Figure 1. The stratum with serum calcidiol ≥70 ng/mL and the stratum with serum calcitriol <10 pg/mL were combined with the next stratum to avoid the bias due to low n (n=3). Number of persons per stratum from left to right: for calcidiol = 164, 357, 253, 122, 53, 18, and 12; for calcitriol = 38, 193, 355, 255, 106, 24, and 8. P by non-adjusted ANOVA. P were considered statistically significant when <0.025 to control for double testing (contrast and linear trend). The line shows the linear trend along strata.

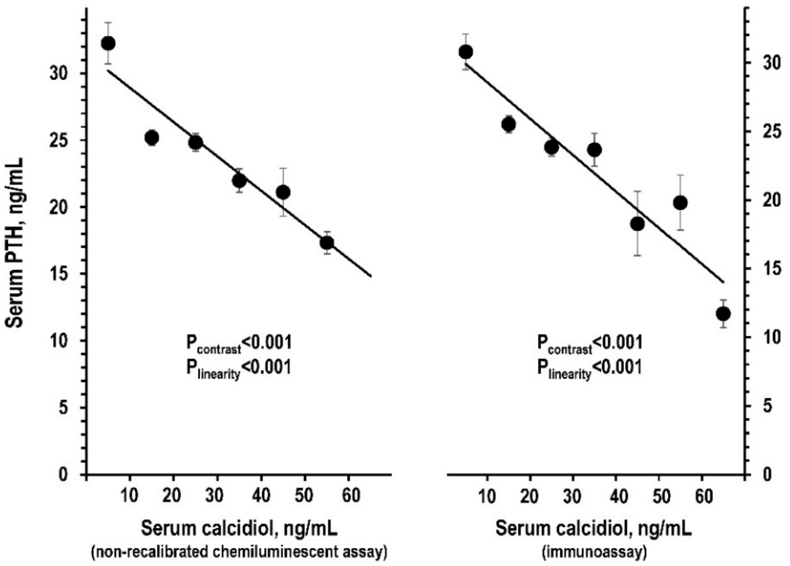

Figure S8. Mean±SE of serum PTH by stratum of recalibrated serum calcidiol (left panel) and of serum calcitriol (right panel) with control for other variables (sex, age, BMI, and eGFR). Strata were defined as in Figure 1. The stratum with serum calcidiol ≥70 ng/mL and the stratum with serum calcitriol <10 pg/mL were combined with the next stratum to avoid the bias due to low n (n=3). Number of persons per stratum from left to right: for calcidiol = 164, 357, 253, 122, 53, 18, and 12; for calcitriol = 38, 193, 355, 255, 106, 24, and 8. P by ANOVA. P were considered statistically significant when <0.025 to control for double testing (contrast and linear trend). The line shows the linear trend along strata.

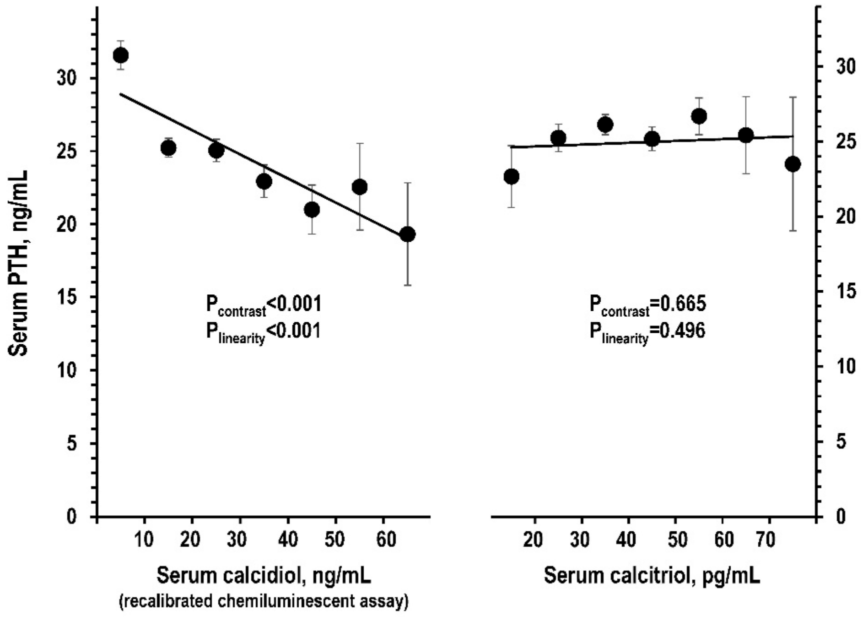

Figure S9. Mean±SE of serum phosphorus (top panels) and of urine phosphorus/creatinine ratio (lower panels) by stratum of serum calcidiol measured by non-recalibrated chemiluminescent assay (left panel) and by immunoassay (right panel). Strata were defined as in Figure 1. The stratum with serum calcidiol ≥70 ng/mL and the stratum with serum calcitriol <10 pg/mL were combined with the next stratum to avoid the bias due to low n (n=3). Number of persons per stratum from left to right: for calcidiol = 164, 357, 253, 122, 53, 18, and 12; for calcitriol = 38, 193, 355, 255, 106, 24, and 8. P by non-adjusted ANOVA. P were considered statistically significant when <0.025 to control for double testing (contrast and linear trend). The line shows the linear trend along strata.

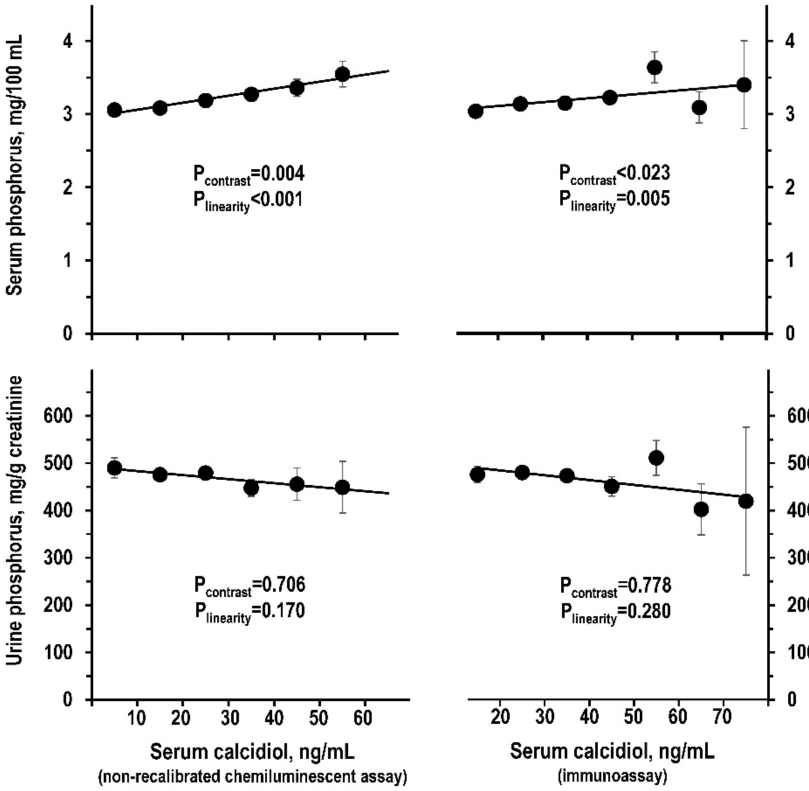

Figure S10. Mean±SE of serum phosphorus (top panels) and of urine phosphorus/creatinine ratio (lower panels) by stratum of recalibrated serum calcidiol (left panel) and of serum calcitriol (right panel) with control for other variables (sex, age, BMI, and eGFR). Strata were defined as in Figure 1. The stratum with serum calcidiol ≥70 ng/mL and the stratum with serum calcitriol <10 pg/mL were combined with the next stratum to avoid the bias due to low n (n=3). Number of persons per stratum from left to right: for calcidiol = 164, 357, 253, 122, 53, 18, and 12; for calcitriol = 38, 193, 355, 255, 106, 24, and 8. P by ANOVA. P were considered statistically significant when <0.025 to control for double testing (contrast and linear trend). The line shows the linear trend along strata.

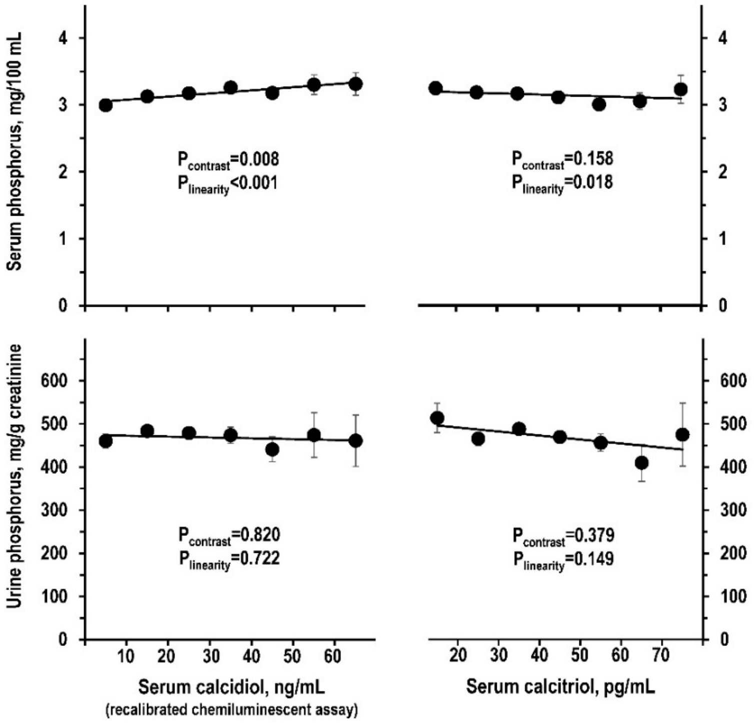

Supplement: Supplementary file 1 [file nutrients-11-01777-s001.pdf]
